# A novel RASA1 mutation causing capillary malformation-arteriovenous malformation (CM-AVM): the first genetic clinical report in East Asia

**DOI:** 10.1186/s41065-018-0062-8

**Published:** 2018-07-16

**Authors:** Ren Cai, Fatao Liu, Chen Hua, Zhang Yu, Michele Ramien, Claudia Malic, Wenxin Yu, Xiaolin Zhang, Yun Liu, Yunbo Jin, Xun Hu, Xiaoxi Lin

**Affiliations:** 1 Department of Plastic and Reconstructive Surgery, Shanghai Ninth People’s Hospital, Shanghai Jiao Tong University School of Medicine, 639 Zhi Zao Ju Road, Shanghai, 200011 People’s Republic of China; 2 Department of Laser and Cosmetic Surgery, Shanghai 9th People’s Hospital, Shanghai, People’s Republic of China; 3 Department of General Surgery, Xinhua Hospital Affiliated to Shanghai Jiao Tong University School of Medicine, Shanghai, People’s Republic of China; 4 Institute of Biliary Tract Disease, Shanghai Jiao Tong University School of Medicine, Shanghai, People’s Republic of China; 5 Department of Dermatology, Children’s Hospital of Eastern Ontario, Ottawa, Canada; 6 Department of Surgery, Children’s Hospital of Eastern Ontario, Ottawa, Canada; 7 Institutes of Biomedical Sciences, Fudan University, Shanghai, People’s Republic of China; 8 Bio-X Institutes, Key Laboratory for the Genetics of Developmental and Neuropsychiatric Disorders (Ministry of Education), Shanghai Jiao Tong University, Shanghai, People’s Republic of China; 9 Department of Stomatology, Luwan Branch of Ruijin Hospital Affiliated to Shanghai Jiao Tong University School of Medicine, Shanghai, People’s Republic of China

**Keywords:** Capillary malformation-arteriovenous malformation, RASA-1 mutation, RasGAP, China, East Asia

## Abstract

Capillary malformation-arteriovenous malformation (CM-AVM) is a clinical entity newly identified in 2003 that is caused by mutation of the RASA-1 gene, which encodes the protein p120-RasGAP. To date, most of the clinical reports on CM-AVM in the literature involve samples entirely consisting of Caucasians of European and North American descent, while reports from China or East Asia are few. Here, we describe a genetic clinical report of CM-AVM. Sequencing revealed a novel stop mutation in the RASA-1 gene causing loss of function (LOF) of the RasGAP domain. To our knowledge, this is the first genetic clinical report of a CM-AVM patient in East Asia. This report may extend our understanding and support further studies of CM-AVM in East Asia.

**Electronic supplementary material:**

The online version of this article (10.1186/s41065-018-0062-8) contains supplementary material, which is available to authorized users.

## Background

Capillary malformations (CMs), or Port-wine stains (PWSs), are a form of vascular anomalies that are congenital, that progressively grow with the individuals, that do not regress spontaneously and that show normal rates of endothelial cell turnover [1–3]. They manifest as erythema that is pink-red in color, and they are related to skin and cutaneous tissue, usually located in the head and nuchal area, and are observed in approximately 3–5 out of every 1000 newborns [4]. In contrast, some atypical CMs occur in cutaneous zones as multiple small, white, pale halos with central red spots that are round to oval in shape [5, 6]. Importantly, these atypical CMs are hereditary in an autosomal dominant fashion, and observation allowed their mapping to the CMC1 locus on chromosome 5q13–22 [7, 8].

In 2003, Eerola et al. reported six families exhibiting this atypical CM, discovered its association with RASA-1 muatation, and defined this disorder as capillary malformation- arteriovenous malformation (CM-AVM) [5]. Given the localized nature of the vascular anomalies, a “second hit” hypothesis was proposed as the pathomechanism of the disorder [5, 6].

In addition to the multiple spots of erythema, fast-flow lesions such as arteriovenous malformation (AVM) or arteriovenous fistula (AVF) as well as hypertrophic syndromes such as Sturge-Weber syndrome (SWS) and Parker-Weber syndrome (PKWS) were also documented in families with a RASA-1 mutation [5, 9]. However, RASA-1 mutation was occasionally discovered in syndromes of overgrowth in length, for example, Klippel-Trenaunay syndrome (KTS) [9, 10].

CM-AVM indicates a high risk of fast-flow lesions. In a 2003 study, 6 out of 17 families manifested with atypical CM together with fast-low lesions such as AVM, AVF or PKWS [5]. Studies in 2008 and 2010 showed that approximately one-third of CM-AVM patients possess fast-flow vascular anomalies [9, 11–13].

RASA-1 encodes for Ras GTPase-activating protein 1, or p120-RasGTP-activating protein (p120-RasGAP), which is a negative regulator catalyzing the conversion of Ras to its GDP-bound form [14].

Mutation of GAP may affect the function of endothelial cells, leading them to form a highly vascularized network and resulting in extensive neuronal death [15]. Stimulation of annexin A6 regulates p120-RasGAP on the plasma membrane and hence controls the activity of Ras [16]. In a murine model experiment, RASA-1^+/−^ mice had a normal phenotype, while homozygous RASA-1^−/−^ mice died at E10.5 from the development of vascular dysfunction and increased neuronal apoptosis [15]. In the literature, most of the RASA-1 mutations associated with CM-AVM are deletions in the reading frame, causing a frame shift and subsequent a formation of a stop codon blocking further translation of the protein. These mutations result in a truncated protein with complete loss of function (LOF) of the RasGAP domain [5].

In this report, we describe two CM-AVM patients with a novel mutation in the RASA-1 gene (c.3070A > T) leading to LOF of the p120-RasGAP domain. To our knowledge, this is the first report of CM-AVM related to a RASA-1 mutation in East Asia.

## Methods

This study was conducted in accordance with the Declaration of Helsinki and was approved by the Ethics Committee of Shanghai Ninth People’s Hospital Affiliated to Shanghai Jiaotong University School of Medicine (equivalent to an institutional review board). This study was performed according to the approved guidelines and regulations. Written informed consent for study participation and publication of identifying information and images was obtained from each subject and child’s guardian prior to the study. A careful clinical record on the proband and his father was obtained by a medical geneticist and plastic surgeons.

## Clinical report

The proband was a boy born at 39 weeks to a 27-year-old G1P1 mother via a eutocia. His birth weight was 3600 g, and his Apgar scores were 9 (0 mins) and 10 (5 mins). Both parents were of Chinese descent without consanguinity. In addition, no prenatal complications were noted by the parents.

**Fig. 1:**
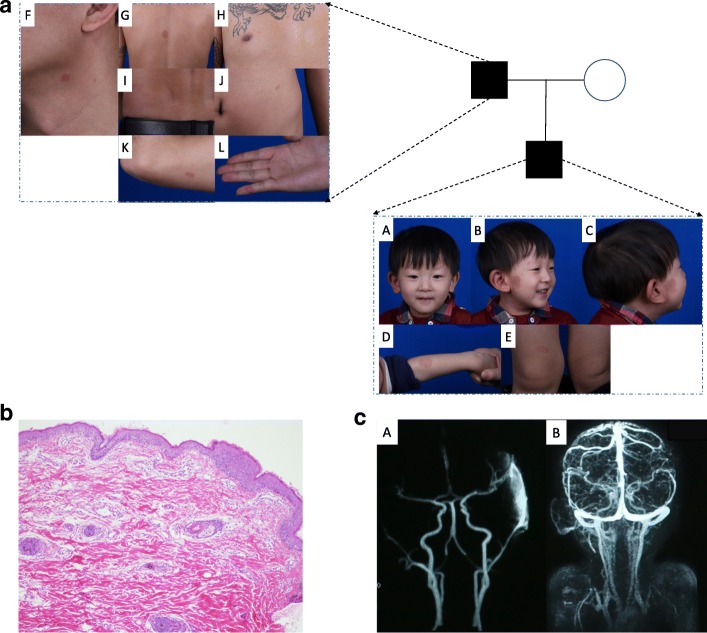
Clinical Manifestations, pathology finds and MRA images of the Proband’s Family. **a**: A: Asymmetric appearance of the ears of the proband. B: Erythema on the head and neck of the proband. C: Erythema on the right parotid area and ear of the probandD: Erythema on the left forearm of the proband. E: Erythema on the right knee of the proband. F: Erythema on the neck of the proband’s father. G: Erythema on the back of the proband’s father. H: Erythema on the chest of the proband’s father. I: Erythema on the waist of the proband’s father. J: Erythema on the abdomen of the proband’s father. K: Erythema on the elbow of the proband’s father. L: Erythema on the right D5 dermatome of the proband’s father. **b**: Hypertrophy in the epidermis and small, tufted capillary malformations in the superficial dermis (100X, HE). **c**: Abnormal arterial networks from the ECA in his right auricular region and neck cutaneous tissue

The proband was born with small red lesions sporadically on his right parotid area. The lesions progressively spread to his right ear region. The skin temperature of the lesion was higher than that of the adjacent normal area. His pulse was detectable with palpation on the lesion. The ears of the proband appeared to be asymmetric (Fig. 1aA). Physical examination revealed multiple spots of erythema in different areas of the body (Additional file 1). He had a large lesion extending from his right cheek to his ear and neck (Fig. 1aB-C). Additionally, there were some oval to round spots of erythema in his left arm and left knee (Fig. 1aD-E). From the family history, the proband’s father had an identical pattern of multiple spots of erythema on the neck, abdomen, back, waist, elbow and right D5 dermatome (Fig. 1aF-L). Multifocal erythema was not observed in other family members.

### Magnetic resonance imaging (contrast-enhanced)

MRI was performed using a 3.0 T MRI unit (MAGNETOM Verio, Siemens, Germany) with a head and neck coil. The MRI protocol included unenhanced axial and coronal T2-weighted sequences, axial T1-weighted sequences, and contrast-enhanced fat-suppressed axial, sagittal and coronal T1-weighted sequences. The parameters for these sequences were as follows: T1-weighted spin echo (SE) sequence = 620-ms repetition time (TR), 8.2-ms echo time (TE), 4-mm section thickness, 260-mm*260-mm field of view, 256*256 matrix; T2-weighted turbo-spin echo sequence = 4500-ms TR, 80-ms TE, 4-mm section thickness, 260-mm*260-mm field of view, 256*256 matrix.

#### DNA extraction

Genomic DNA was extracted from peripheral blood by using a QIAamp DNA Blood Mini Kit (QIAGEN) according to the manufacturer’s instructions. A skin biopsy was performed on the CM-AVM lesion of the proband via a standard procedure. DNA of biopsy tissues was extracted by using the QIAamp DNA Mini Kit (QIAGEN) with overnight incubation in proteinase K at 56°C for approximately 14 h. A Qubit 2.0 fluorimeter (Life Technologies, Carlsbad, CA, USA) and a Thermo NanoDrop 2000 spectrophotometer (Thermo, Wilmington, DE, USA) were used to determine the DNA concentration and quality.

#### Next-generation sequencing (NGS) and Bioinformatic analysis

To target the exons as well as the corresponding boundary regions, a series of RNA baits for RASA-1 were designed and synthesized. We used the SureSelectXT kit reagents (Agilent Technologies, Santa Clara, CA) for the Illumina adapters. The biotinylated RNA baits were applied in hybridization reactions at 65°C for 24 h to generate the adapter-ligated DNA libraries. Then, streptavidin-coated magnetic beads were used to capture the hybridized DNA targets. The target DNA was washed, eluted, and finally barcoded. An Agilent 2100 Bioanalyzer (Thermo) was used to determine the length and integrity of the library.

Quantitative PCR (KAPA Biosystems, Wilmington, MA) was applied to verify the concentration of the indexed libraries, after which the libraries were sequenced on a MiSeq instrument (Illumina, San Diego, CA). The resulting sequencing reads were trimmed with known adaptors, quality-checked and then mapped to a human reference genome (hg19, downloaded from http://genome.ucsc.edu) by using Burrows-Wheeler Aligner (BWA 0.5.9, http://bio-bwa.sourceforge.net) with the default parameters. PCR duplicates were marked and removed by using PICARD software. Additionally, Genome Analysis Toolkit (GATK, https://www.broadinstitute.org/gatk/) was applied to conduct indel realignment, base recalibration and variant calling. The resulting .vcf files were then annotated by VEP software (http://www.ensembl.org/info/docs/tools/vep/) and the GEMINI framework (http://gemini.readthedocs.io/en/latest/index.html). By using Integrative Genomic Viewer (IGV) software, variants were visualized and checked.

#### Sanger sequencing

RASA1 exon 25 was PCR-amplified to track the c.3070A > T (p.Lys1024*) variant in the blood and tissue samples from the family. Primer sequences are available upon request. Amplicon fragments were sequenced bidirectionally (forward and reverse) with the M13 primer by the BigDye Terminator v3.1 cycle sequencing kit and an ABI 3730 DNA Analyzer (Life Technologies, Carlsbad, CA). Target sequences were compared to the RASA1 reference sequence (NM_002890) using Mutation Surveyor (SoftGenetics, State College, PA) (Additional file 2).

## Results

**Fig. 2:**
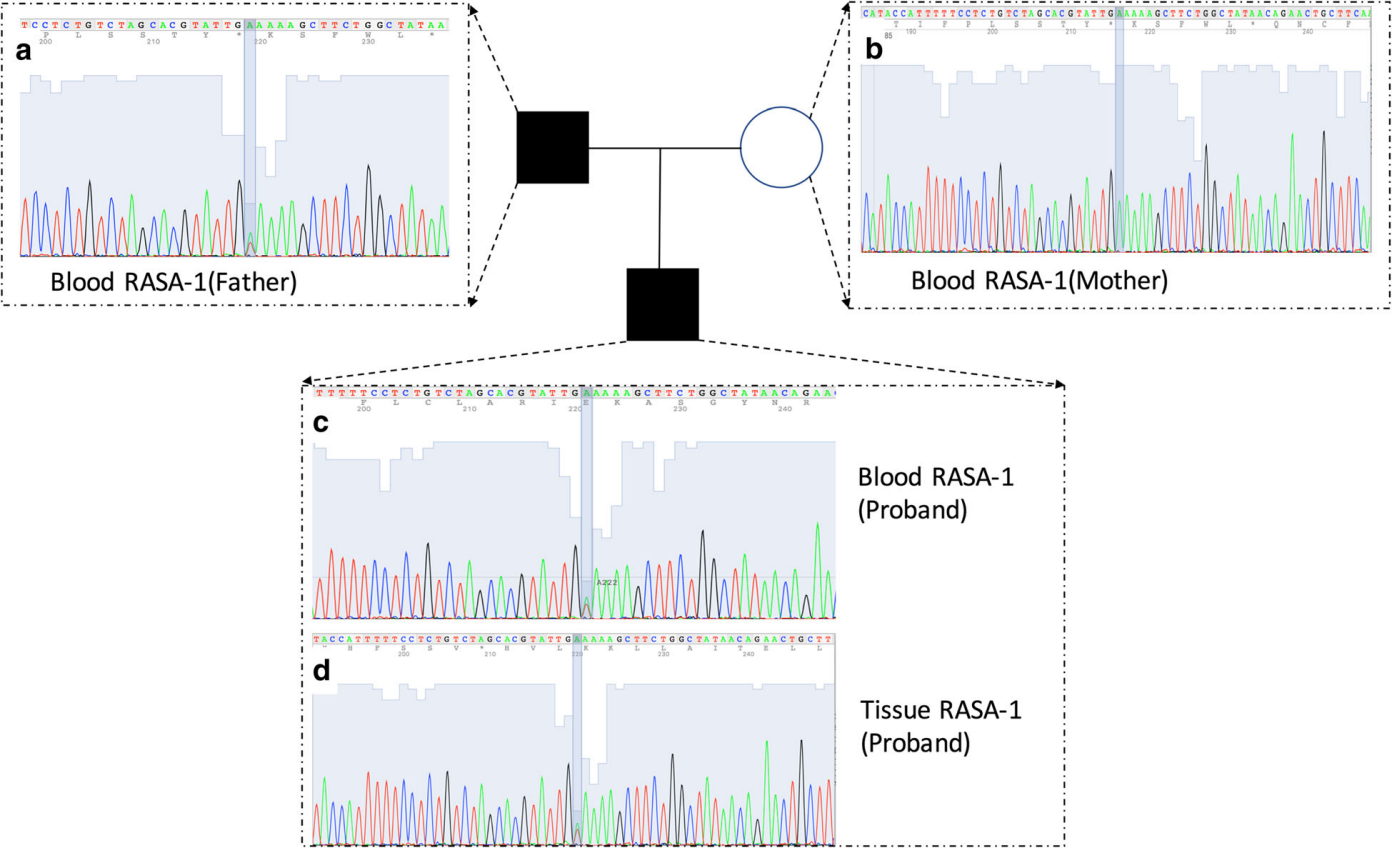
Sanger Sequencing of the RASA-1 mutation of the DNA blood sample of the proband, the proband’s parents and DNA tissue sample of the proband. Sanger sequencing of the RASA-1 mutation using DNA from the blood sample of the proband and the proband’s parents and the tissue sample of the proband

**Fig. 3:**
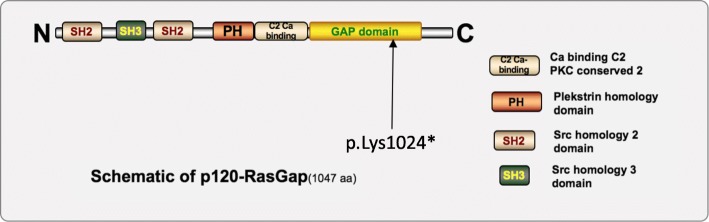
Schematic of p120-RasGAP. Schematic of p120-RasGAP, the 1047-amino-acid protein encoded by the RASA-1 gene. With two Src homology 2 (SH2) domains and one Src homology 3 (SH3) domain in the N-terminal region, a Pleckstrin homology (PH) domain and a protein kinase conserved region 2 in the central region (C2), and a Ras GTPase-activating domain in the C-terminal region (RasGAP), p120-GAP negatively regulates the Ras/MAPK pathway. Our patient and his father harbored a mutation (c.3070A > T, p.Lys1024*), labeled with the solid arrow, halting the further translation of p120-RasGAP, which consequently led to CM-AVM

Molecular analysis of the DNA extracted from the peripheral blood of the proband and the proband’s parents confirmed that the proband’s father and the proband harbored a familial germline mutation (c.3070A > T, p.Lys1024*) in the RASA-1 gene (Figs. 2 and 3). NGS analysis showed a depth of coverage for this mutation of 16,260× and 16,451× in blood samples (Table 1). This mutation was also detected in the proband’s skin lesion at a depth of coverage of 21,026× (Table 1). The score of this RASA-1 germline mutation (c.3070A > T, p.Lys1024*) from the 1000 Genomes database of East Asia was − 1, indicating that the mutation had never been reported before.

**Table 1:** NGS Genetic Information of mutation of proband and proband's father

DNA Sample	Blood of the Proband’s Father	Blood of the Proband	Tissue of the Proband
gene	RASA1	RASA1	RASA1
chr:posi	5:86686626	5:86686626	5:86686626
ref > alt	A > T	A > T	A > T
type	SNV	SNV	SNV
geno	0/1	0/1	0/1
AD	8787 + 7664	8491 + 7769	10,029 + 10,997
hgvs_c	c.3070A > T	c.3070A > T	c.3070A > T
hgvs_p	p.Lys1024*	p.Lys1024*	p.Lys1024*
impact	stop gained	stop gained	stop gained
impact_severity	HIGH	HIGH	HIGH
aaf_1kg_eas	None	None	None
in_exac	None	None	None

Radiologic Assessment: Contrast-enhanced MRI demonstrated that there were abnormal arterial networks from the external carotid artery (ECA) in his right auricular region and neck cutaneous tissue (Fig. 1c). Because of the harm of radiation, we did not perform digital subtraction angiography (DSA)on the child (Fig. 1cA-B).

Pathologic Findings: Under the microscope, hyperplasia was observed in the epidermis showed hyperplasia, small tufted vessels were observed in the superficial dermis, and ectatic vessels were observed in the deep dermis (Fig. 1b, HE, 100x).

## Discussion

The proband and his father were diagnosed with CM-AVM based on their clinical manifestations according to the diagnostic criteria [17]. Suspicions of CM-AVM were raised due to the multifocal cutaneous erythema observed even without the fast-flow lesions [13]. Findings of congenital and acquired skin lesions that were round to oval in shape and 1–3 cm in diameter and that had small anemic halos confirmed the disorder at the clinical level [5, 6, 18, 19]. In addition, the LOF mutation of RASA-1 enabled a definite diagnosis at the genetic level. MRI demonstrated abnormal networks from the ECA, which indicated that the patients had the potential for AVM invasion.

According to the literature, CM-AVM patients have a high risk of high-flow lesions such as AVM and AVF [9, 11–13], which can affect soft tissues such as dermis, muscle and even central nervous system tissues, leading to anomalies, malformations, dysfunction or disabilities [20]. In this case, according to the results of MRI, the patient had the potential for AVM invasion. Precautions such as regular clinical follow-up and bleomycin injection should be taken to prevent AVM formation [21].

RASA1 facilitates the inactivation of Ras p21 by enhancing the GTPase activity of Ras proteins [22]. LOF mutation of RASA-1 may have numerous consequences on downstream signaling, such as aberrant proliferation, angiogenesis [23] and increased ERK activation in vitro [24]. The mutation of RASA-1 in this case was the base substitution c.3070A > T (p.Lys1024*, Fig. 3) present in the RasGAP domain halting further translation, leading to the LOF of the RASA1 gene.

The association between the phenotype and the genotype of the RASA-1 mutation in CM-AVM is still unclear. The genotype-phenotype relationship and its mechanisms have not been well elucidated, even though there are some hypothesized mechanisms, such as changes in the expression pattern of different growth factors due to the RASA-1 mutation [5]. Another proposal is the somatic “second-hit” hypothesis, in which fast-flow lesions may form because of haploinsufficiency of the RASA-1 mutation when a specific population of cells experience complete LOF caused by a “second-hit” somatic mutation in addition to the “first-hit” germline mutation [6, 11].

However, in this case, did we not detect any additional mutations on the RASA-1 gene to support the “second-hit” hypothesis, nor did we detect a somatic mutation of GNAQ, which is observed in 88% of SWS cases and 92% of apparently nonsyndromic PWS cases [25]. Thus, we can differentiate CM-AVM from nonsyndromic PWSs at the genetic level. The specific cell type harboring the mutation is not known and still requires further study.

The phenotype of this CM-AVM family was discordant. The proband exhibited multifocal erythema and potential AVM invasion from the ECA, while his father exhibited multifocal erythema without any high-flow lesions. We do not know whether there was intrafamilial variability in RASA-1 mutation-related conditions in this family, and further study is needed to clarify this issue.

This study emphasizes the importance of NGS, together with a careful medical history and good cooperation with the radiology department, for facilitating the discovery of new clinical findings. As plastic surgeons or dermatologists, we can easily overlook or neglect multifocal erythema and choose laser therapy as the treatment, which may lead to a great risk when the erythema is part of a CM-AVM lesion with underlying AVM. The proband will receive genetic testing; know the potential risk of fast-flow lesion formation; have regular clinical follow-ups to prevent lesion formation or obtain appropriate initial treatment; and take precautions against the life-threating complications of AVM or AVF, such as hemorrhage, congestive heart failure, and seizures [5, 11, 12, 17].

## Conclusion

The mutation of RASA-1 in this patient is the base substitution c.3070A > T (p.Lys1024*) present in the RasGAP domain halting further translation. To our knowledge, this is the first description of this mutation in East Asia. It would be valuable to measure the Ras/MAPK activity level in vitro, such as in HUVECs from the samples collected from the patient and his father. Such information would help us understand the angiogenesis of CM-AVM, especially for patients in East Asia.

## Additional files

Additional file 1: Cutaneous findings of the proband’s family. (DOC 37 kb)

Additional file 2: Information about the primers used to detect the c.3070A > T (p.Lys1024*) variant. (DOC 35 kb)

## Abbreviations

AVF: Arteriovenous Fistula
AVM: Arteriovenous Malformation
CM: Capillary Malformation
CM-AVM: Capillary Malformation-Arteriovenous Malformation
ECA: External Carotid Artery
KTS: Klippel-Trenaunay Syndrome
LOF: Loss-of-Function
MRI: Magnetic Resonance Imaging
PKWS: Parker-Weber Syndrome
PWS: Port-Wine Stain
SWS: Sturge-Weber Syndrome
