# Supplementary material for: A novel RASA1 mutation causing capillary malformation-arteriovenous malformation (CM-AVM): the first genetic clinical report in East Asia
Source: Hereditas. 2018 Jul 16;155:24. doi: 10.1186/s41065-018-0062-8 (PMC6048896; doi:10.1186/s41065-018-0062-8)
Supplement: Supplementary file 1 — Cutaneous findings of the proband’s family. (DOC 37 kb) [file 41065_2018_62_MOESM1_ESM.doc]

| **Lesions** | **PARCITIPANT** | **LOCATION** | **Small/Large** | **Color** | **CM** | **AVM** |
| --- | --- | --- | --- | --- | --- | --- |
| 1 | S | Right ear | Large | Dark Red | Yes | Yes |
| 2 | S | Right cheek | Large | Dark Red | Yes | No |
| 3 | S | Right Neck | Large | Dark Red | Yes | Yes |
| 4 | S | Left forearm | Small | Pink | Yes | No |
| 5 | S | Right knee | Small | Pink | Yes | No |
| 6 | F | Left Neck | Small | Pink | Yes | No |
| 7 | F | Back | Small | Red | Yes | No |
| 8 | F | Waist | Large | Dark red | Yes | No |
| 9 | F | Chest | Small | Red | Yes | No |
| 10 | F | Abdomen | Small | Red | Yes | No |
| 11 | F | Right Elbow | Small | Red | Yes | No |
| 12 | F | Right D5 | Small | Pink | Yes | No |
