# Supplementary material for: A novel RASA1 mutation causing capillary malformation-arteriovenous malformation (CM-AVM): the first genetic clinical report in East Asia
Source: Hereditas. 2018 Jul 16;155:24. doi: 10.1186/s41065-018-0062-8 (PMC6048896; doi:10.1186/s41065-018-0062-8)
Supplement: Supplementary file 2 [file 41065_2018_62_MOESM2_ESM.doc]

Seq ID	Orientation	Seq
RASA-1-5:86686626	FORWARD	TAGGAGCATGCCAAACACAA
RASA-1-5:86686626	REVERSE	TGGAATGTGGAAAAGTGTGC
